# An abbreviated therapy-dosimetric equation for the companion diagnostic/therapeutic [^64/67^Cu]Cu-SARTATE

**DOI:** 10.1186/s13550-021-00814-6

**Published:** 2021-08-21

**Authors:** Eric Laffon, Henri de Clermont, Roger Marthan

**Affiliations:** 1 CHU de Bordeaux, 33000 Bordeaux, France; 2 Univ. Bordeaux, Centre de Recherche Cardio-Thoracique de Bordeaux, 33000 Bordeaux, France; 3 INSERM U-1045, Centre de Recherche Cardio-Thoracique de Bordeaux, 33000 Bordeaux, France; 4 Service de Médecine Nucléaire, Hôpital du Haut-Lévèque, Avenue de Magellan, 33604 Pessac, France

**Keywords:** Theranostics, SARTATE, Dosimetry, Cumulated activity, Neuroblastoma

## Abstract

In a preclinical model of neuroblastoma, Dearling et al. recently demonstrated the potential interest for a theranostic approach of [^64^/^67^Cu]Cu-SARTATE for the detection and treatment of SSTR2-positive neuroblastoma lesions in pediatric patients whose widespread metastases survive initial therapy as minimal residual disease (MRD). MRD may be detected by [^64^Cu]Cu-SARTATE and subsequently treated by [^67^Cu]Cu-SARTATE. Since therapeutic dosimetry estimation of the latter agent from the uptake of the former one in the initial diagnostic scan was not addressed, the present theoretical commentary proposes the derivation of an abbreviated therapy-dosimetric equation for the companion diagnostic/therapeutic [^64^/^67^Cu]Cu-SARTATE that might be of interest for future clinical theranostic practice.

## Background

Companion diagnostic/therapeutic radiopharmaceuticals (D-RP/T-RP) are the basis of the theranostic strategy that uses a molecule designed for a specific target, which is labeled with a suitable pair of radionuclides [1, 2]. The strategy consists in a first diagnostic PET-imaging scan with D-RP that selects patients who can subsequently benefit from a therapy with T-RP. It therefore appears instrumental to predict cumulated activity ($\tilde{A}_{{\text{C}}}$), and, hence, delivered radiation-dose of T-RP in tumors, from D-RP uptake in the initial diagnostic scan. In this connection, a model was previously proposed for comparing kinetic parameters and $\tilde{A}_{{\text{C}}}$ of the companion ^64^Cu/^177^Lu-cetuximab [2].

Recently, the [^64^Cu]Cu-SARTATE biodistribution was assessed by Dearling et al. in a preclinical intrahepatic model of neuroblastoma (NB) metastatic disease, representing minimal residual disease (MRD), along with potential therapeutic effect of [^67^Cu]Cu-SARTATE [1]. Unlike the companion ^64^Cu/^177^Lu-cetuximab, whose input function (IF, i.e., RP-blood time-activity-curve) was different for diagnosis/therapy (time constant of mono-exponentially decaying IF of 0.0830 h^−1^ and 0.0224 h^−1^, uncorrected for physical decay, respectively), the companion [^64^/^67^Cu]Cu-SARTATE provides the opportunity of using a “true” theranostic pair of radionuclides, resulting in identical D-RP/T-RP uptake features [1, 2].

The present theoretical commentary aims at deriving an abbreviated equation of therapeutic $\tilde{A}_{{\text{C}}}$ with [^67^Cu]Cu-SARTATE, from [^64^Cu]Cu-SARTATE uptake assessed in a single initial diagnostic scan. In the pre-clinical framework of Dearling et al., this equation was refined by using the common mouse IF of [^64/67^Cu]Cu-SARTATE and the physical-decay-rate constant of the ^64/67^Cu radionuclides [1].

## Methods

### Derivation

Assuming irreversible trapping, when PET imaging is acquired at peak time of decay-uncorrected trapped-tracer-activity concentration, it has been shown that [3]:

1 $${\text{SUV}}_{{{\text{Tumor}}}} (t_{{\text{peak - uncorr}}} )/{\text{SUV}}_{{{\text{Blood}}}} (t_{{\text{peak - uncorr}}} ) = K_{i} /\lambda + F$$

where SUV_Tumor_ and SUV_Blood_ (g mL^−1^) are the mean standard-uptake-value in tumor and blood, respectively, *K*_*i*_ (mL time^−1^ mL^−1^) is the tracer uptake-rate constant in tumor, *λ* (time^−1^) is the tracer physical-decay-rate constant and *F* (mL mL^−1^) is the fraction of free tracer in blood and interstitial volume (i.e., Patlak-plot y-intercept).

Furthermore, the total number of disintegrations $\tilde{A}_{C}$ (i.e., cumulated activity; Bq s) occurring in tissue volume *V* (mL) after tracer injection can be estimated as [2, 4]:

2 $$\tilde{A}_{C} = V \times [\tilde{A}\_{\text{IF}}] \times [(K_{i} /\lambda) + F]$$

where $[\tilde{A}\_{\text{IF}}]$ (Bq s mL^−1^) is the area under curve of the tracer decay-uncorrected IF.

Applying Eq. 1 to D-RP leads to:

3 $$K_{i - T} = k \times \lambda_{D} \times [{\text{SUV}}_{{{\text{Tumor - }}D}} (t_{{\text{peak - uncorr}}} )/{\text{SUV}}_{{{\text{Blood-}}D}} (t_{{\text{peak - uncorr}}} ){-}F_{D} ]$$

where *k* = *K*_*i*-*T*_/*K*_*i*-*D*_, *λ*_*D*_ is the physical-decay-rate constant of [^64^Cu]Cu-SARTATE and F_D_ is its free fraction in blood and interstitial volume.

Incorporating Eq. 3 into Eq. 2 applied to T-RP yields the general equation of its cumulated activity:

4 $$\tilde{A}_{C - T} = V \times \left[ {\tilde{A}\_{\text{IF}}_{ - T} } \right] \times [k \times (\lambda_{D} /\lambda_{T} ) \times [{\text{SUR}}_{D} (t_{{\text{peak - uncorr}}} ){-}F_{D} ] + F_{T} ]$$

where λ_T_ is the physical-decay-rate constant of [^67^Cu]Cu-SARTATE, F_T_ is its free fraction in blood and interstitial volume, and SUR_D_(t_peak-uncorr_) is the tumor-to-blood SUV ratio (no unit) assessed at peak time of decay-uncorrected activity concentration of trapped D-RP [5].

### Input function of [^64^/^67^Cu]Cu-SARTATE and peak time

Mean blood-clearance data provided by Dearling et al. were used to fit the common IF of [^64/67^Cu]Cu-SARTATE, after removing decay correction (GraphPad Prism 6 software) [1]: IF(*t*) = *Y*_0_ × exp(−*α* × *t*) with *Y*_0_ = 4.11939 %IA/g (IA: injected activity in Bq) and *α* = 0.18934 h^−1^ (n = 4; *R* = 0.999; *P* < 0.01; 95%-CI of 4.08542–4.15335 and of 0.18579–0.19289, respectively). As a result, $[\tilde{A}\_{\text{IF}}_{{\text{-T}}} ]$ involving [^67^Cu]Cu-SARTATE was 783 × IA mL^−1^ (= [*Y*_0_ × IA × 3600]/[100 × *α*]), assuming tissue density of 1 g mL^−1^.

Peak time of decay-uncorrected activity concentration of trapped [^64^Cu]Cu-SARTATE was assessed from an analytical solution of the nonlinear Patlak’s equation, involving the above-reported mono-exponentially decaying IF [6]:

5 $$\begin{aligned}C_{{{\text{Trapped - }}D}} {(}t) & = Y_{0} \times K_{i - D}  \\ & \quad \times [\exp ({-}\alpha \times t){-}\exp ({-}\lambda_{D} \times t)]/[\lambda_{D} {-}\alpha]\end{aligned}$$

Peak time could thus be graphically determined, and, when solving Eq. 5 for dC_Trapped-*D*_(*t*)/*dt* = 0, could be alternatively computed as *t*_peak-uncorr_ = Log(*α*/λ_D_)/(*α* − λ_D_). Furthermore, SUR_D_(t_peak-uncorr_) (= SUV_Tumor-D_(t_peak-uncorr_)/SUV_Blood-D_(t_peak-uncorr_)) in Eq. 4 was obtained by adding the term “F_D_ × IF(t_peak-uncorr_)” to Eq. 5 right-hand side and by dividing the whole by IF(t_peak-uncorr_), since the tumor-to-blood SUV ratio equals the tumor-to-blood activity–concentration ratio [6].

## Results

**Fig. 1:**
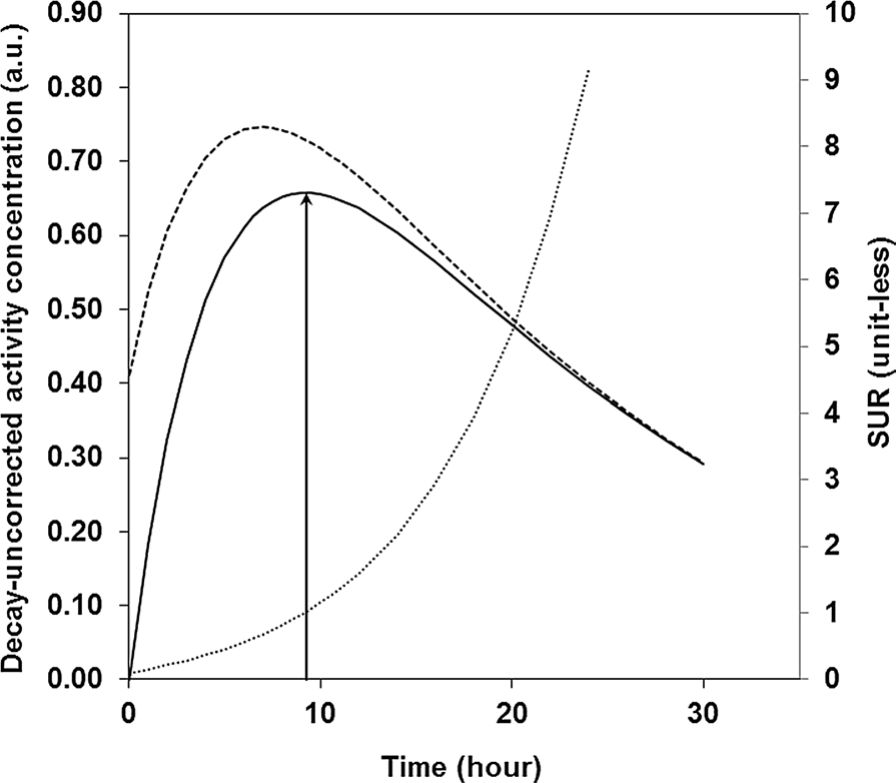
Decay-uncorrected activity concentration versus time of trapped (full) and of tumor (dashed) [^64^Cu]Cu-SARTATE; SUR versus time (dotted). Peak time of decay-uncorrected trapped-tracer activity concentration is indicated

Figure 1 shows the decay-uncorrected activity concentration of trapped and tumor [^64^Cu]Cu-SARTATE versus time (in arbitrary unit) that were obtained from Eq. 5. The trapped-tracer time-activity curve (TAC; in arbitrary unit) was drawn by setting an arbitrary K_i-D_ value of 0.05 mL h^−1^ mL^−1^, which does not play a role in determining its peak time (Eq. 5) [2, 4, 6]. The trapped-tracer-TAC peak time could thus be graphically assessed at 9 h post-injection, coherently with the computed outcome of t_peak-uncorr_ = Log(*α*/λ_D_)/(*α* *−* λ_D_) [2, 6]. The t_peak-uncorr_ computing emphasizes the [^64/67^Cu]Cu-SARTATE IF time constant *α*, whose 95%-CI limits obtained from fitting Dearling et al.’s mean blood-clearance data yielded a 1.1%-relative change in the peak-time value (corresponding to a 6-min-absolute change). In comparison with the trapped-tracer TAC, the tumor TAC additionally involves free tracer in blood and interstitial volume, of which fraction F_D_ was arbitrarily set to 0.1 mL mL^−1^. Figure 1 also shows the corresponding SUR-versus-time curve. At *t* = 9 h post-injection, the SUR value is close to 1 (for the above *K*_*i*-D_ and F_D_ values), thus indicating that tumor- and blood-activity concentration are close, of about 27 × 10^3^ Bq g^−1^ (for a mean [^64^Cu]Cu-SARTATE IA of 3.61 × 10^6^ Bq [1]). An ± 1-h uptake-time variability around peak time results in a + 19/− 17% increase/decrease in SUR, respectively.

Since the companion [^64^/^67^Cu]Cu-SARTATE uses a “true” theranostic pair of radionuclides, resulting in chemically identical Cu-labeled SARTATE molecules, it is then assumed that *k* = 1 (i.e., *K*_*i*-T_ = *K*_*i*-D_) and *F*_D_ = *F*_T_ = *F*. As a consequence, an refined abbreviated equation of therapeutic [^67^Cu]Cu-SARTATE $\tilde{A}_{C}$, from [^64^Cu]Cu-SARTATE uptake in a diagnostic scan achieved at *t*_peak-uncorr_ = 9 h post-injection, is:

6 $$\tilde{A}_{{C - 67{\text{Cu}}}} = 783 \times V \times IA \times [4.87 \times {\text{SUR}}_{{ - 64{\text{Cu}}}} (t = 9h){-}3.87 \times F]$$

where *λ*_D_/*λ*_T_ = 4.87.

## Discussion

In a pre-clinical intrahepatic model of NB metastatic disease representing MRD, Dearling et al. measured the biodistribution of [^64^Cu]Cu-SARTATE and evaluated the potential of [^67^Cu] Cu-SARTATE as a therapeutic agent [1]. In this framework, Eq. 6 allows computing of an estimate of [^67^Cu]Cu-SARTATE $\tilde{A}_{{\text{C}}}$, and, hence, of delivered radiation-dose, involving the SUR assessed in an initial [^64^Cu]Cu-SARTATE diagnostic scan acquired at 9 h post-injection. The assumptions made for deriving Eq. 6 are justified since [^64^/^67^Cu]Cu-SARTATE provides the opportunity of using a “true” theranostic pair of radionuclides for which (i) *K*_*i*-T_ = *K*_*i*-D_ (same irreversible trapping) (ii) *F*_D_ = *F*_T_ (same fraction of free tracer in blood and interstitial volume) and (iii) same IF.

The SUR increases with time (Fig. 1), and, consequently, after/before peak time of 9 h post-injection**,** the SUR value involved in Eq. 6 is over/underestimated, respectively. *A* ± 1-h uptake-time variability around peak time results in a + 19/− 17% increase/decrease in SUR, and, hence, in an over/underestimation of [^67^Cu]Cu-SARTATE $\tilde{A}_{{\text{C}}}$, respectively. However, Hofheinz et al. have shown in human [^18^F]-FDG PET imaging that correcting SUR for uptake time to an arbitrary value may lead to reduced test–retest variability in comparison with that of the SUV [7]. In this connection, we suggest that, potentially, correcting SUR for uptake time (to 9 h post-injection in the current mouse framework) might provide an [^67^Cu]Cu-SARTATE $\tilde{A}_{{\text{C}}}$ value with reduced test–retest variability.

The accuracy of the blood-activity-concentration measurements plays a critical role in the measurement uncertainty of [^67^Cu]Cu-SARTATE $\tilde{A}_{{\text{C}}}$. Since *t*_peak-uncorr_ = Log(*α*/*λ*_D_)/(*α* − λ_D_), inaccurate blood-activity-concentration measurements result in an inaccurate estimate of the time constant of the [^64/67^Cu]Cu-SARTATE IF (i.e., of *α*), and, hence, in a biased estimate of the peak-time value, leading then to the above-addressed over/underestimation of [^67^Cu]Cu-SARTATE $\tilde{A}_{{\text{C}}}$. Furthermore, assuming *t*_peak-uncorr_ is accurately known, an under/overestimation of [^64^Cu]Cu-SARTATE blood-activity concentration in a mouse at peak time results in an increase/decrease in SUR, and, hence, in an over/underestimation of [^67^Cu]Cu-SARTATE $\tilde{A}_{{\text{C}}}$, respectively. More precisely, the absolute change in [^67^Cu]Cu-SARTATE $\tilde{A}_{{\text{C}}}$ may be assessed from Eq. 6, as:

7 $$\Delta \ A_{{C - 67{\text{Cu}}}} = 783 \times V \times IA \times 4.87 \times (1/f{-}1) \times {\text{SUR}}_{{-64{\text{Cu}}}} (t = 9h)$$

where *f* is the factor of either under- or overestimation of [^64^Cu]Cu-SARTATE blood-activity concentration in a mouse at peak time. However, a convolutional neural network has been recently investigated in humans that can provide robust automatic image-based mean values of [^18^F]-FDG SUV_Blood_ over the aorta. We thus suggest that such a device might also be relevant in mouse [^64^Cu]Cu-SARTATE PET imaging to reduce the measurement uncertainty of SUV_Blood_(t_peak-uncorr_), and, hence, that of [^64^Cu]Cu-SARTATE SUR(t_peak-uncorr_) and of [^67^Cu]Cu-SARTATE $\tilde{A}_{{\text{C}}}$ [8].

Equation 6 might be further simplified by using a mean value for F, obtained from experiments that remain to be performed. When F is considered negligible compared to SUR_-64Cu_ (*t* = 9 h), an overestimate of $\tilde{A}_{{{\text{C-}}67{\text{Cu}}}}$ is provided, that is more acceptable than an underestimate for therapeutic purpose: the higher the [^64^Cu]Cu-SARTATE uptake, the higher the peak-time SUR_-64Cu_ and the less significant the overestimation.

The scope of Eq. 6 is limited to the pre-clinical intrahepatic model of NB metastatic disease representing MRD, since mouse data published by Dearling et al. were used [1]. Therefore, the current theoretical commentary should be considered as a pre-clinical step for determining whether [^64^Cu]Cu-SARTATE imaging might reliably predict dosimetry with [^67^Cu]Cu-SARTATE and, hence, might predict therapeutic outcome of patients in future clinical theranostic practice. Indeed, clinical translation requires additional experiments in pre-clinical models, followed by experiments in humans to investigate the measurement uncertainty of the patient-specific [^64/67^Cu]Cu-SARTATE IF involved in t_peak-uncorr_ and in $[\tilde{A}\_{\text{IF}}_{{\text{-T}}} ]$, respectively, as well as that of the [^64^Cu]Cu-SARTATE SUR possibly uptake-time corrected to t_peak-uncorr_.

## Conclusions

The companion [^64^/^67^Cu]Cu-SARTATE provides the opportunity of using a “true” theranostic pair of radionuclides, that allows deriving an abbreviated equation of therapeutic [^67^Cu]Cu-SARTATE $\tilde{A}_{{\text{C}}}$. This equation emphasizes the [^64^Cu]Cu-SARTATE SUR assessed in a single diagnostic scan acquired at peak time of decay-uncorrected activity concentration of trapped [^64^Cu]Cu-SARTATE. We suggest that it might be of interest for future clinical theranostic practice.

## Data Availability

The datasets used and/or analyzed during the current study are available from the corresponding author on reasonable request.

## Abbreviations

$$\tilde{A}_{{\text{C}}}$$: Cumulated activity
$[\tilde{A}\_{\text{IF}}]$: Area under curve of the tracer decay-uncorrected input function
D-RP/T-RP: Diagnostic/therapeutic radiopharmaceuticals
*F*: Fraction of free tracer in blood and interstitial volume
IF: Tracer input function
*Ki*: Tracer uptake-rate constant in tumor
*λ*: Tracer physical-decay-rate constant
MRD: Minimal residual disease
NB: Neuroblastoma
SUVBlood: Mean standard-uptake-value in blood
SUVTumor: Mean standard-uptake-value in tumor
SUR: Tumor-to-blood SUV ratio
TAC: Time–activity curve
*t*peak-uncorr: Peak time of decay-uncorrected trapped-tracer activity concentration
PET: Positron emission tomography
